# Association of Health Status and Nicotine Consumption with SARS-CoV-2 positivity rates

**DOI:** 10.1186/s12889-021-11867-6

**Published:** 2021-10-03

**Authors:** Thomas J. Duszynski, William Fadel, Kara K. Wools-Kaloustian, Brian E. Dixon, Constantin Yiannoutsos, Paul K. Halverson, Nir Menachemi

**Affiliations:** 1 Indiana University Richard M. Fairbanks School of Public Health, Indianapolis, USA; 2 Indiana University School of Medicine, Indianapolis, USA; 3 Regenstrief Institute, Inc., Indianapolis, USA

**Keywords:** Smoking, Health status, COVID-19, SARS-CoV-2

## Abstract

**Background:**

Much of what is known about COVID-19 risk factors comes from patients with serious symptoms who test positive. While risk factors for hospitalization or death include chronic conditions and smoking; less is known about how health status or nicotine consumption is associated with risk of SARS-CoV-2 infection among individuals who do not present clinically.

**Methods:**

Two community-based population samples (including individuals randomly and nonrandomly selected for statewide testing, *n* = 8214) underwent SARS-CoV-2 testing in nonclinical settings. Each participant was tested for current (viral PCR) and past (antibody) infection in either April or June of 2020. Before testing, participants provided demographic information and self-reported health status and nicotine and tobacco behaviors (smoking, chewing, vaping/e-cigarettes). Using descriptive statistics and a bivariate logistic regression model, we examined the association between health status and use of tobacco or nicotine with SARS-CoV-2 positivity on either PCR or antibody tests.

**Results:**

Compared to people with self-identified “excellent” or very good health status, those reporting “good” or “fair” health status had a higher risk of past or current infections. Positive smoking status was inversely associated with SARS-CoV-2 infection. Chewing tobacco was associated with infection and the use of vaping/e-cigarettes was not associated with infection.

**Conclusions:**

In a statewide, community-based population drawn for SARS-CoV-2 testing, we find that overall health status was associated with infection rates. Unlike in studies of COVID-19 patients, smoking status was inversely associated with SARS-CoV-2 positivity. More research is needed to further understand the nature of this relationship.

## Background

Early data from China identified smokers as overrepresented among severely ill COVID-19 patients and smoking appeared to be a significant risk factor for severe infection and adverse outcomes [1]. Comorbid conditions (which are also more common among smokers), and increased age were first identified as risk factors for severe disease and death in China [2–5]. Data from other countries also showed an increased risk of death with each additional decade of life [6, 7]. Poor health status and the presence of immunocompromising conditions each emerged as predictive indicators of severe or fatal outcomes [8, 9]. Among the more than 7000 cases of COVID-19 initially reported in the U.S., more than 37% had at least one underlying health condition that would be considered a risk factor for severe disease, hospitalization, and intensive care with mechanical ventilation [9]. Importantly, at the beginning of the pandemic, most of the existing epidemiological information was based on those with severe disease because asymptomatic and mildly symptomatic individuals are not routinely tested in the United States nor many other countries.

Thus, it is unknown how specific health behaviors and overall health status are associated with SARS-CoV-2 infection, including mild infection, as opposed to moderate-to-severe cases, which have resulted in hospitalization or death. Smoking, vaping, and other uses of nicotine and tobacco products are significant risk factors for multiple poor health outcomes in a population including tissue damage and cancers [10–13]. Both smoking and vaping can weaken lung tissue and be a source of inflammation potentially creating an opportunity for infection to occur [12, 14]. However, while it is known that tobacco use is associated with severe disease, it is less clear if such behaviors are associated with an increased risk of infection [15]. In sum, the relationship between nicotine consumption and risk of infection is unclear and being evaluated in other studies globally [16].

Our study builds upon a large statewide community sample (including random and nonrandomly selected individuals) that were tested by the state health department in Indiana for both active viral infection and the presence of antibodies for SARS-CoV-2 [17]. Specifically, in the current study we examine how overall health status, as well as nicotine consumption and smoking behaviors, are associated with current and previous infection with SARS-CoV-2. Indiana has higher adult and youth smoking rates than the US; and the use of electronic cigarettes and vaping products nearly doubled in the past 6 years [14, 18]. As such, our data represents an opportunity to understand the epidemiology of key risk factors especially among asymptomatic and mildly symptomatic individuals not included in previous research. Understanding who is most at risk for infection is paramount in the ability to control the spread and protect those at highest risk until vaccines are widely distributed worldwide.

## Methods

In the current study, we pool data from two statewide testing efforts where participants were tested for active viral infection and the presence of antibodies for SARS-CoV-2 in Indiana. The pooled data represented two separate waves that were part of a statewide prevalence study conducted April 25–29 and June 3–7, 2020 which used identical sampling strategies and methods. In each wave, some participants were randomly selected based on Indiana records from tax year 2019 and 2018 to create a de-duplicated list of Indiana residents that included those who filed or co-filed taxes and all of their dependents. Updated contact information was then merged from the Indiana Bureau of Motor Vehicles and other state databases. In an effort to address under-representation of minorities in statewide testing efforts, each wave also included outreach and open testing to a nonrandom group of racial and ethnic minorities. In each wave, the random sample was stratified by each of the state’s 10 public health preparedness districts representing children and adults aged 12 years or older. In all cases, excluded individuals were those that were institutionalized, had an out-of-state address, or did not meet age eligibility (< 12 years of age).

Randomly selected participants were contacted through postcards, text messages, email, and telephone and given information to either register online or speak to a live operator [19]. Nonrandom outreach was done in conjunction with civic, religious, and community leaders in both the African American and Hispanic communities, to identify neighborhood centers where underserved populations could be tested. All individuals in both the random and nonrandom groups of both waves are analyzed herein to examine how health status and behaviors are associated with SARS-CoV-2 infection. This pooled group represents community-based individuals who were tested regardless of symptoms, medical indications, risk factors, and prior testing history—and is thus different than all other hospital or clinic-based groups previously studied.

Prior to testing, participants were asked to complete a form which included questions about demographic characteristics, self-reported health status, and their primary nicotine and tobacco consumption behaviors. These behaviors included cigarette, chewing tobacco or vaping usage and responses were categorized as every day, somedays, or not at all, consistent with the Behavioral Risk Factor Surveillance System (BRFSS) questionnaire [19].

Trained personnel used Dacron swabs to collect nasopharyngeal specimens for RT-PCR testing for SARS-CoV-2. RT-PCR testing was conducted using the Lilly Clinical Diagnostics Lab SARS-CoV-2 test based on the CDC primary set, the Luminexon NxTAG CoV Extended Panel, or the Roche cobas SARS-CoV-2 test depending on platform availability. A venipuncture was used to obtain 2–3 mL of blood for antibody testing using a chemiluminescent microparticle immunoassay for detection of SARS-CoV-2 IgG [20]. At the time of the study, these assays received emergency use authorization and no formal test of sensitivity or specificity were possible due to the lack of a gold standard. Nevertheless, laboratory-based negative percent agreement was 100% for all tests and positive percent agreement was 90% for one RT-PCR test and 100% for the others. The antibody test used has an estimated 100% sensitivity (at 14 days after symptom onset) and a specificity of 99.6% [17].

### Statistical analysis

All analyses were performed using R (version 3.6.3) on the combined samples from wave 1 and 2 of the prevalence study. Descriptive statistics were calculated for the sample population using frequencies and percentages. A bivariate logistic regression model controlling for age, sex, race, ethnicity, and whether a person was randomly selected or in an outreach sample to examine the association between both health status and use of nicotine with SARS-CoV-2 positivity on either PCR or antibody tests. The effects of sampling group and household clustering were controlled for using random effects in the model.

The Institutional Review Board (IRB) at Indiana University deemed the study as exempt from human subjects research under the public health surveillance exemption.

## Results

Including both random and nonrandom samples, from two distinct waves of testing, the total sample size for this study was 8214 individuals whose demographic characteristics are displayed in Table 1. Among five possible categories, 16% self-reported being in “excellent” health while “very good” and “good” were reported by 38 and 35%, respectively.

**Table 1:** Demographic characteristics of the sample including self-reported health status and tobacco use (*n* = 8214)

Characteristics	Participants (%)
Female	4565 (55.6)
Male	3647 (44.4)
White	6599 (80.3)
Non-white	1615 (19.7)
Hispanic	653 (7.9)
Non-Hispanic	7561 (92.0)
Age: < 40	2379 (28.9)
Age: 40–59	3036 (36.9)
Age: 60+	2799 (34.0)
Randomly selected group	
Wave 1	3658 (44.5)
Wave 2	2668 (32.5)
Nonrandomly selected group	
Wave 1	937 (11.4)
Wave 2	951 (11.6)
**Self -reported Health Status**	
Excellent	1331 (16.2)
Very Good	3140 (38.2)
Good	2894 (35.2)
Fair	699 (8.5)
Poor	92 (1.1)
**Cigarette Use**	
Everyday	656 (7.9)
Some days	224 (2.7)
Not at all	7276 (88.6)
**Current chewing tobacco use**	
Everyday	115 (1.4)
Some days	79 (0.9)
Not at all	7962 (96.9)
**Current vaping or e-cigarette use**	
Everyday	110 (1.3)
Some days	126 (1.5)
Not at all	7920 (96.4)

There were more than 1300 persons who identified as using tobacco either every day or on some days. Almost 11% of respondents reported current cigarette use including 656 (7.9%) who smoke every day and 224 (2.7%) who smoked on some days. Reported use of chewing tobacco was less frequent with 115 (1.4%) reporting using chewing tobacco daily, while 79 (0.9%) chewed on some days. Vaping or e-cigarettes use was reported by 2.8% including 110 individuals who reported vaping daily (1.3%) and 126 individuals who reported vaping on some days (1.5%).

The bivariate relationship between tobacco use and SARS-CoV-2 positivity rates (PCR and antibody) are displayed in Table 2. Increased frequency of cigarette use was inversely associated with current infection, previous infection, and overall population prevalence. For example, whereas the population prevalence among respondent who do not currently smoke cigarettes was 6.6%, the population prevalence among those that report smoking everyday was 2.0% (*p* < 0.001).

**Table 2:** Bivariate Infection rates and prevalence by tobacco use

Tobacco/Smoking Behaviors	Current infection %	Previous infection %	Population prevalence (e.g., Current or Previous Infection) %	*P*-Values
Current cigarette Use				
➝ Every day	1.2%	0.8%	2.0%	< 0.001
➝ Some days	4.5%	2.7%	7.2%	
➝ Not at all	4.9%	1.8%	6.6%	
Current chewing tobacco use				
➝ Every day	6.1%	0.8%	7.0%	0.121
➝ Some days	9.2%	2.6%	11.8%	
➝ Not at all	4.5%	1.7%	6.2%	
Current vaping or e-cigarette use				
➝ Every day	2.8%	0%	2.7%	0.329
➝ Some days	3.2%	3.2%	6.4%	
➝ Not at all	4.6%	1.7%	6.3%	

In the logistic regression model (see Table 3), compared to individuals self-reporting excellent health status, those reporting good (OR = 1.42, 95% CI: 1.05–1.91) or fair (OR = 1.78, 95% CI: 1.23–2.57) health status had significantly higher positivity rates. Those who reported “poor” health status did not have higher positivity rates, however they represented only 1.1% of the sample. With respect to nicotine consumption, whereas smoking cigarettes (daily or on some days) was inversely associated with infection rates (OR = 0.49, 95% CI: 0.32–0.74); the use of chewing tobacco products was positively associated with infection (OR = 2.17, 95% CI: 1.26–3.72). The use of vaping/e-cigarettes was not associated with infection rates (OR = 0.654, 95% CI: 0.32–1.35).

**Table 3:** Binary logistic regression, controlling for age, sex, race, ethnicity, and randomized or non-randomized sample examining relationship between infection and self-reported health status and tobacco use

	Odds Ratio	95% Confidence Intervals	*P-*Value
Excellent Health Status	Reference	Reference	Reference
Very Good Health Status	1.02	0.75–1.39	0.896
Good Health Status	1.42	1.05–1.91	0.022
Fair Health Status	1.78	1.23–2.57	0.002
Poor Health Status	1.83	0.82–4.07	0.137
Any Cigarette Smoking	0.49	0.32–0.74	< 0.001
Chewing Tobacco	2.17	1.26–3.72	0.005
Vaping/e-cigarettes	0.654	0.32–1.35	0.250
Male	1.04	0.85–1.26	0.715
White	0.30	0.24–0.38	< 0.001
Hispanic	6.22	4.74–8.17	< 0.001
Age Group < 40	Reference	Reference	Reference
Age Group 40–59	0.77	0.61–0.96	0.022
Age Group 60+	0.44	0.33–0.60	< 0.001

## Conclusions

As self-reported health status declined, the risk of being infected increased in a community-based population undergoing active viral and seroprevalence testing in nonclinical settings. Importantly, those in poor health status might not have been able to participate in the study resulting in an unknown risk of infection in this population. The use of cigarettes, especially daily, was inversely associated with infection. This finding is biologically plausible given the growing body of evidence that nicotine has an effect on the angiotensin-converting enzyme (ACE 2) [21]. Though the literature is complex and somewhat inconsistent, nicotine may bind with the ACE 2 receptor, which is associated with a reduction in ACE 2 levels in multiple organs. Thus, nicotine may reduce the number of binding sites available to SARS-CoV-2, which also binds to the ACE 2 receptor [20]. Nicotine use may create a cellular response that may increase the number of receptors on a cell surface in the lungs and could be protective against tissue damage from SARS-CoV-2 infection [22].

However, previous research suggests that those who smoke and become infected are more likely to have severe disease and overall poor outcomes as documented in an early study from China, in which more smokers had more severe disease requiring hospitalization and mechanical ventilation as compared to those who did not smoke [23]. In the same study, smokers were more likely to die as compared to non-smokers [23]. Our findings provide a new view of the relationship between smoking and SARS-CoV-2 infections but is consistent with the growing body of evidence that found similar inverse associations between smoking and hospitalization for COVID-19, which has been documented in other countries [23, 24]. As with other studies, we lacked granular data on the duration and/or quantity of nicotine intake underpinning the observed associations. We do however know that comorbid conditions associated with severe disease and death such as heart disease, diabetes, obesity, cerebrovascular disease, hypertension, COPD or other lung conditions, kidney disease are all associated with or worsened by smoking [25].

This is the first population-based study to assess behavioral risk factors for SARS-CoV2 infection. As previously noted, data on the cumulative quantity and duration of cigarette smoking, and detailed previous nicotine or tobacco history is lacking so it is not possible to identify how much exposure is needed that lead to this inverse association. In addition, because our study did not collect data on individuals that were institutionalized, generalizing to these and other excluded groups should be done with caution.

The current study found an inverse epidemiological association between smoking and infection with SARS-CoV-2 that is contrary to findings from predominantly severely ill COVID-19 patients. More study is needed to better understand the complex interaction between smoking, SARS-CoV-2 infection, and adverse clinical outcomes.

## Data Availability

The datasets used and/or analyzed during the current study are available from the corresponding author on reasonable request.
